# Supplementary material for: Are carbon emissions trading and green financial instruments synergistic? -Comprehensive quantitative research based on content analysis
Source: PLoS One. 2024 Mar 7;19(3):e0298601. doi: 10.1371/journal.pone.0298601 (PMC10919601; doi:10.1371/journal.pone.0298601)
Supplement: S1 Data — (ZIP) [file pone.0298601.s001.zip › Data/地级市Z_F工作报告.zip!/╣π╢1⁄2/╒╪╟∞╩╨/└┤╫╘╣1⁄2╓┌║┼í╛┬φ┐╦╩2╛▌═°í┐_2016.docx]

各位代表：

　　我代表肇庆市人民政府向大会作工作报告，请予审议，并请政协委员和其他列席人员提出意见。

经济社会发展取得新成效 “十二五”目标任务顺利完成

　　过去一年，面对经济下行压力加大的严峻挑战，在省委、省政府和市委的正确领导下，在市人大和市政协的监督支持下，市政府紧紧围绕建设珠三角连接大西南枢纽门户城市的目标，全面落实《珠三角规划纲要》，深入实施“两区引领两化”战略，主动适应经济新常态，着力稳增长、促改革、调结构、惠民生、防风险，基本完成市十二届人大五次会议确定的目标任务。全年实现地区生产总值1970亿元，比上年增长（以下简称增长）8.2%，人均生产总值48670元，增长7.7%，一、二、三产业增加值分别增长4.4%、8.5%、9.2%，规模以上工业总产值突破4千亿元，农林牧渔业增加值增速排名全省第一。完成一般公共预算收入143.4亿元，增长1.2%。完成固定资产投资1330亿元、社会消费品零售总额632.4亿元、外贸进出口总额82.1亿美元，分别增长16.8%、12.9%、4.8%，分别高于全省增速1、2.8和9.8个百分点，其中社会消费品零售总额增速排名全省第二、珠三角第一，外贸进出口总额增速排名珠三角第一。全体居民人均可支配收入为18991.4元，增长9.6%，其中城镇和农村常住居民人均可支配收入分别为23746元和13982元，分别增长9.3%和10.6%。“暖企行动”共减免企业税负约15.8亿元，减免涉企行政事业性收费、基金收费约1.6亿元。肇庆高新区被纳入珠三角国家自主创新示范区，肇庆新区新增批准融资规模34.3亿元、新增到位资金20亿元。我市成为国家物流标准化试点城市和广东省首批小微企业创业创新基地城市示范。新竣工投产的招商引资项目96个，新引进计划投资共757亿元的项目126个、项目履约率达76%。完成交通建设投资121亿元，广佛肇城际轨道建成通车，38项省重点项目完成年度计划投资的124%。向上争取财政扶持资金158亿元，增长38%。年末全市实际融资余额1665.6亿元，存贷比为71.8%，位居全省第三。争取到国家发改委对我市项目资本金专项基金11.8亿元，居全省地级市首位，高出地级市平均水平3.8亿元。高要撤市设区，我市再次被确认为国家卫生城市。空气质量达标天数比上年增加57天，PM2.5年均浓度下降幅度名列全省第二、珠三角第一，PM10年均浓度下降幅度名列全省第一，空气质量综合指数在全省排名比上年上升2位。

　　实体经济保持平稳增长。实施支持实体经济发展20条措施等系列政策，协调解决企业反映的用地、用工、税费、资金等方面260多个困难问题，全年取消45项、停征11项行政事业性收费，免征42项小微企业行政事业性收费等一批税费。设立1.1亿元市级中小微企业贷款风险补偿基金，年末全市实际融资余额增长16.6%，其中本地银行表内、表外、外地银行投放在我市的融资余额分别增长9.3%、26.6%和103%。全市完成规模以上工业增加值938.2亿元，企业利润总额增长12.5%，新增规模以上工业企业68家、纳税超千万元企业12家，新登记市场主体及其注册资本金分别增长23.5%和6.7%，新增6家企业在新三板挂牌。

　　重点项目建设提速推进。大力开展“三抢”（抢建、抢搭、抢种）专项整治行动，累计清拆面积近91万平方米。全年获批棚户区改造贷款27亿元。推广运用PPP项目12个，总投资约199亿元。广佛肇高速大旺至封开江口段累计完成总投资的77.4%，怀阳高速肇庆段先行工程、汕昆和汕湛高速肇庆段全线动工建设，西江航道界首至肇庆大桥段扩能升级工程启动实施，肇庆—南沙“无水港”试航成功。唯品会华南运营中心、中国人寿电子商务有限公司华南区域中心、中油天然气有限公司液化天然气等产业项目建成投产。100项市重点项目完成投资410.8亿元，超额完成年度投资计划。

　　产业结构加快调整升级。设立1亿元市级创新驱动发展引导资金，争取到国家和省科技专项资金1.1亿元、增长4.2倍，深化与清华大学、广东省科学院等知名院校的战略合作，组建了华南师范大学（肇庆）国际光电产业研究院和华农（肇庆）生物产业技术研究院，有75家企业列入省高新技术企业培育计划，新增国家级科技企业孵化器培育单位2家，新创名牌产品20个、驰名商标2个。装备制造业、高技术产业投资分别增长45.3%和204.4%，规模以上装备制造企业增加到285家、其中新增6家。工业技改投资增长41.6%，10家企业实施机器人应用。肇庆三榕电子商务产业园被评为国家电子商务示范基地，限额以上批零企业通过网络实现商品零售额增长53.2倍。对外贸易转型升级加快，全市离岸服务外包执行额增长40.6%，高于全省平均水平15.4个百分点。旅游标准化建设稳步推进，旅游收入增长9.3%。粮食生产实现“七连增”，70%乡镇建成农产品质量安全检测站，率先在全省搭建农产品电子商务服务平台。

　　平台园区实现扩能增效。成功举办“肇庆金秋”、汽配产业、环保产业和北京、广州、佛山、深圳、珠海等多场招商会，全年实际吸收外资13.9亿美元，增长4.6%，比全省平均水平高4.6个百分点。肇庆高新区新动工项目20个，新建成投产项目26个，引进了4个投资超10亿元项目，有2家企业创税超亿元。粤桂合作特别试验区（肇庆）加快“六个一”基础设施建设，新引进项目10个。广佛肇（怀集）经济合作区完成“七通一平”，已建成投产项目18个，新引进项目11个。肇庆（高要）汽车零部件产业园新增入园企业15家。肇庆大旺、德庆、怀集3个省级产业园和高要、四会、广宁3个产业集聚地实现工业增加值242.8亿元、全口径税收21.5亿元，分别增长12.1%和19.5%。

　　城乡建设取得新突破。制定实施“三规合一”工作方案，启动修编新一轮城市总体规划。市辖区面积扩大3倍，中心城区规划范围从194平方公里扩大到563平方公里。肇庆新区全年完成重点项目投资45.5亿元，已建成或在建道路12条共25.2公里，引进了投资超百亿的启迪环保科技城和中国优质农产品交易中心等重点产业项目。省运会31个市政配套项目全面铺开，大冲枢纽立交等城市化道路改造工程动工建设，阅江大桥完成总投资的67%。“巩卫创文”有效推进，启动建设数字化城市管理平台，强力整治城市管理突出问题。完成农村环境连片整治年度任务，新创建省卫生镇4个、省卫生村122条，完成16条生态村、80条名村、123条示范村创建任务。

　　环境保护建设扎实有效。设立了肇庆西江保护管理和综合利用委员会、粤桂联合环境监控预警及应急指挥中心，开展“环境法治年”及环境保护大检查活动，“零容忍”查处环保违法行为，全年开展了5次市内跨区域联合执法行动，在全省率先执行按日处罚政策，全面完成省下达的污染物排放总量控制目标任务。开展大气污染防治四大战役专项行动，淘汰黄标车及老旧车辆19782辆，空气质量一级优天数增长102.6%，PM2.5、PM10年均浓度分别下降25%和24.3%。市第三污水厂二期项目以及一批沿江镇、工业园区污水处理厂建成使用，西江肇庆段水质优于Ⅱ类标准，集中式饮用水源地水质100％达标。新建成森林公园23个，森林覆盖率提高到70.3%。耕地保有量增加到283.8万亩，连续15年实现耕地占补平衡。

　　社会民生事业蓬勃发展。一般公共预算用于民生类支出191.4亿元，增长12.3%，占比达71.5%。省、市民生实事全面完成。城镇新增就业近5万人，城镇登记失业率低于2.6%的控制目标。实现全国义务教育发展基本均衡县全覆盖，普通高中市一级以上优质学校比例达90.9%，教师学生创作和使用教育信息资源全省排名第一。市文化发展中心动工建设，包公文化园和端砚文化村建成开放，94.2%的乡镇（街道）综合文化站创建成为省一级站。全市新增保障性住房4338套（户），完成省下达任务的104%。有效防控登革热等重大传染病，县级公立医院综合改革实现全覆盖，乡镇卫生院“五个一”医疗设备配备全面完成。各项社会保险待遇发放率保持100%。企业退休人员月基本养老金提高到1876元，实现“十一年连涨”，城乡居民养老保险基础养老金提高到每人每月100元。基本医保与大病保险实现一站式结算，城镇职工、城乡居民医保年度最高支付额分别达到55万元和27万元，城乡特困居民医疗救助人均补助标准提高到1556元以上。东南板块低保标准实现城乡一体化。60岁以上老人免费乘坐城市公交车和进入市内国营景区。第十四届省运会金牌数量位列全省第四，第十五届省运会筹备工作全面启动。完成“六五”普法任务。食品药品安全、安全生产、社会治安形势稳定。统计、民族、宗教、外事、侨务、港澳、对台、人防、国防动员、地方志、档案、气象、水文、地震、妇女儿童、残疾人、慈善等工作取得新成绩。

　　政府自身建设不断强化。严格按照法定制度向市人大及其常委会报告工作、向市政协通报情况，按期办结人大代表建议和政协提案。依法行政工作定期报告、重大行政决策专家咨询论证和合法性审查、政府法律顾问等制度进一步落实，全面推行行政复议开庭审理、公开听证等阳光工程。13个试点单位2332项行政职权全面公开，市直单位权责清单基本编制完成，不再保留“非行政许可审批”事项。“三严三实”专题教育和“机关优作风抓落实年”活动扎实开展，突出整治不作为、慢作为、乱作为等懒政庸政怠政懈政现象。惩治和预防腐败体系建设更加健全，厉行节约反对浪费和审计监督等制度得到强化落实，《市民问政》等社会媒体监督不断加强，全市党政机关公务用车制度改革顺利完成。

　　各位代表！过去一年全市经济社会发展取得新成效，标志着“十二五”发展各项目标任务顺利完成。

　　五年来，我们强化落实“两区引领两化”战略，区域综合实力不断增强。肇庆高新区被认定为国家知识产权试点园区，经济总量增长1.5倍，规模以上工业增加值增长1.3倍；肇庆新区中央绿轴生态城成为国家绿色生态示范城区，累计获批融资78亿元、实际到位55.4亿元，征储土地2.8万亩。“两化”步伐加快，全市工业总产值实现从2000亿到4000亿的“三级跳”，规模以上工业增加值年均增长17.8%，工业化向中期迈进；“一江两岸”超百万人口城市框架初步搭建，各地新城区初具规模。经济实力进一步提升，生产总值比“十一五”期末接近翻一番，年均增长11.1%；人均生产总值年均增长10.1%；一般公共预算收入年均增长14.1%；固定资产投资、社会消费品零售总额、外贸出口总额分别年均增长19.8%、14.9%和13%；累计实际吸收外资61.5亿美元，比“十一五”增长48.4%。

　　五年来，我们大力推进交通基础设施建设，枢纽门户城市功能逐步完善。完成交通基础设施建设投资比“十一五”增长98.6%，建成或在建轨道交通、高速公路共10条，轨道交通、高速公路通车里程分别新增252公里和101.7公里，先后迈进“高铁时代”和“城轨时代”，高铁通车里程全省居首。东引西连合作发展功能不断加强，分别与广佛、梧州合作推进59个和48个项目，粤桂合作特别试验区成为我国首个东西部省际合作示范区，广佛肇经济合作区上升为省级平台。

　　五年来，我们积极实施创新驱动，转型升级步伐持续加快。对比“十一五”期末，全社会研发投入增长2.3倍，省级以上各类创新研发平台和高新技术企业分别新增50家、74家，发明专利申请量增长2.1倍，高新技术产品产值占比提高9个百分点，被评为全国科技进步考核先进市。金属加工产业成为首个年产值超千亿产业集群，年主营业务收入超10亿元企业数量是“十一五”期末的5倍，被列入珠江西岸先进装备制造产业带。现代服务业增加值占服务业增加值比重提高到45.2%。农业龙头企业比“十一五”期末增加81家。三次产业比重调整为14.7:49.2:36.1。单位建设用地二、三产业增加值“十二五”期末比期初增长36.6%。节能减排各项任务全面完成。

　　五年来，我们突出抓好重点领域改革，发展活力不断提升。农村综合改革、“多规合一”等多项国家和省改革试点落户我市。各类金融机构增至86家，存贷比比“十一五”期末提高11个百分点。市县政府机构改革全面完成，在全省率先实行网上联合审批服务，审批事项精简率达40%以上，行政审批实际办结时限比法定时限平均提速74%。建成省网上办事大厅肇庆分厅，实现省市县镇村五级连通。新企业设立实现“一表登记、三证同发”。建成全国首创行政审批“阳光通、便民通”短信服务平台。152条行政村完成“村改居”，县镇村“三资”管理平台实现全覆盖。

　　五年来，我们切实增进民生福祉，发展成果更多惠及人民群众。城镇和农村常住居民人均可支配收入分别年均增长11.3%和13.6%。一般公共预算投入民生类支出比“十一五”增长177.9%。创建成为省教育强市，普及从学前教育到高中阶段十五年教育。建立起统一覆盖常住人口的城乡居民基本养老和基本医疗保险制度。保障房建设和棚户区改造数量比“十一五”增长3.5倍。城镇、农村低保补助标准比“十一五”期末翻一番。完成农村公路硬底化2147公里。蝉联全国社会治安综合治理优秀市“长安杯”。两轮“双到”扶贫共帮扶18.8万贫困人口和347条贫困村脱贫，佛山市和中山市对我市扶贫开发给予了大力支持。

　　各位代表！过去五年发展成果来之不易，这是省委、省政府和市委总揽全局、正确领导的结果，是市人大、市政协有效监督、大力支持的结果，是全市人民和社会各界凝心聚力、团结奋斗的结果。我代表市政府，向全市人民，向驻肇部队和各级驻肇单位，向所有关心支持参与肇庆建设发展的各界人士致以衷心的感谢！

　　同时，我们也清醒认识到，我市经济社会发展仍然存在不少困难和问题：去年生产总值、规模以上工业增加值、一般公共预算收入等指标增幅未能实现预期目标，人均生产总值与全国、全省同期水平还有差距，发展基础薄弱、经济总量偏小、经济结构不优、质量水平不高等问题仍未能得到根本解决；工业集聚创新能力有待增强，六大高耗能产业产值占比达37.6%，现代服务业发展滞后，大部分产业仍处于中低端，转型升级任务艰巨；产城融合互动还不够紧密，城市经营管理水平有待提高，城镇化率仍然低于全国、全省平均水平；民生建设存在“短板”，教育、医疗、社会保障等公共服务均等化水平还不高，城乡居民收入水平有待进一步提升；环境综合治理效果与人民群众的期望还有差距，空气质量不佳的问题仍未根本解决，空气质量综合指数在全省排名仍然靠后；懒政庸政怠政懈政现象依然存在，少数领导干部和公务员奋发有为精神不足，执行力有待提高，政府自身建设仍需加倍努力。我们将本着对全市人民高度负责的态度，采取更有针对性措施，破解发展难题，推动跨越发展。

主动适应经济新常态 全力推动“十三五”追赶型发展

　　“十三五”时期是我市全面建成小康社会的决胜期，是建设枢纽门户城市的加速期。我国确立了创新、协调、绿色、开放、共享五大发展理念，坚持稳中求进工作总基调，推进供给侧结构性改革，经济基本面将保持长期向好。我省深入实施创新驱动战略，强力推进珠三角优化发展和粤东西北地区振兴发展，努力在显著提高质量效益的基础上实现经济持续平稳发展。在新的历史起点上，市委十一届七次全会描绘了我市“十三五”发展的宏伟蓝图，确立了以五大发展理念为指引实施追赶型发展的总体思路，突出把全面建设小康社会、加快建设枢纽门户城市作为追赶型发展的核心任务。我们既要清醒看到，全球经济形势复杂多变，国内“三期叠加”影响依然存在，经济下行压力仍将持续，我市破解经济发展结构性矛盾、适应资源环境刚性约束、加快产业转型升级的要求尤为紧迫，同时更要看到，我市作为“一带一路”的重要节点和“三大经济带”交汇点，享有珠三角和粤东西北地区“双重”政策支持，后发优势前所未有，仍然处于可以大有作为的战略机遇期。只要我们坚定信心不气馁，咬定目标不旁移，狠抓发展不松劲，真抓实干不空谈，就一定能够实现追赶型发展的新跨越。

　　我市“十三五”总体发展要求和主要预期目标：全面贯彻党的十八大和十八届历次全会精神，深入贯彻习近平总书记系列重要讲话精神，牢固树立创新、协调、绿色、开放、共享发展理念，按照我省“三个定位、两个率先”的目标要求，围绕建设珠三角连接大西南枢纽门户城市的核心任务，深入实施“两区引领两化”战略，着力推进供给侧结构性改革，全力推进总量规模与质量效益同步提升、东南板块与山区板块相互促进、经济与环境互为包容、自身与外部良性互动、全体居民参与分享的追赶型发展，确保与全省同步率先全面建成小康社会，加快建设珠三角连接大西南的枢纽门户城市。力争到2020年，全市地区生产总值达到3000亿元左右，年均增长9%左右；一般公共预算收入、规模以上工业增加值、固定资产投资、社会消费品零售总额分别年均增长10%、11.5%、18%、10.5%；城乡居民人均可支配收入年均增长9%；单位GDP能耗、主要污染物排放量控制在省下达指标内，空气质量优良率达到90%以上。“十三五”时期，我们将重点抓好以下工作：

　　——推进追赶型发展，率先全面建成小康社会。树立发展新标杆，努力保持中高速增长，力争经济增速高于珠三角和全省平均水平，确保2018年达到全面小康各项指标要求，2020年全面小康总体水平与全省平均水平差距进一步缩小。构建发展新格局，统筹实施两大板块分类指导，力争东南板块人均GDP尽快达到珠三角平均水平，山区板块人均GDP尽快达到全国平均水平。增创发展新优势，深入推进重点领域改革攻坚，加快转变政府职能，努力构建市场化、法治化、国际化投资营商环境，激发“大众创业、万众创新”活力。

　　——强化功能建设，加快构建枢纽门户城市。全面落实枢纽门户城市建设五年行动计划，大力实施“八大工程”。着力提升交通通行力，协同建设一批对接大西南、珠三角其他城市和粤东西北的高快速公路、铁路新干线，实现县县通铁路，建成“内环—外环”高速路网和东南板块组团之间的快速路网，改造提升航道网，推进枢纽客货运站场建设，构建起市域各重点区域点对点快捷联通、珠三角连接大西南畅通发达的综合交通枢纽。着力加快新型城市化，把肇庆新区打造成为枢纽门户城市核心载体，实现“八年建新城”；统筹推进“一江两岸三区”组团式建设，推动城市空间架构沿西江主轴展开；加快推进西江、绥江城镇带扩容提质，力争全市城镇化率提高到55%。提高城市宜居性和可持续性，积极建设海绵城市，确保2017年创建成为全国文明城市。着力扩大对外开放度，协同共建广佛肇+清云韶经济圈，深化与大西南、港澳台、“一带一路”国家和地区合作，努力打造国家东西部产业合作示范区、珠江—西江经济带区域中心城市、粤桂黔高铁经济带先行区。坚持以大招商招大商为开放合作主线，突出以优质项目带动增量、提升存量，强化平台园区扩能增效，力争五年引进投资900亿元，2020年实际吸收外资达16亿美元。

　　——实施创新驱动，积极打造现代产业体系。坚持产业兴市、工业强市，力促产业迈向中高端。着力提升创新能力，以肇庆高新区为核心加快建设珠三角国家自主创新示范区重要节点，强化落实“西江人才计划”，力争研发机构覆盖25%以上规模工业企业，高新技术企业数量、研发经费支出占GDP比重、发明专利申请量和授权量均比2015年翻一番，高技术产品产值占规模以上工业总产值比重达38%。着力推动主导产业集聚发展，加快肇庆高新区现代科技工业城建设，构建“一县一核心园区一特色主导产业”发展新格局，发展壮大先进装备制造业，培育发展高端新型电子信息、新材料、生物医药、节能环保和新能源产业，力争形成产值超千亿产业集群2—3个、超500亿产业集群3—4个。着力加快传统优势产业转型升级，打好工业转型升级攻坚战，发展与文化、健康、生态深度融合的大旅游，促进现代农业提速增效。积极发展新经济、新业态，加快建设“宽带肇庆”、“智慧城市”，力争现代服务业占服务业增加值比重提高到47.2%。

　　——守住生态环保底线，努力实现“绿富同兴”。绝不以透支资源来换取一时的效益，绝不以牺牲环境来换取一时的繁荣，绝不以牺牲老百姓的健康和子孙后代的福祉来换取一时的政绩，保护绿水青山蓝天，打造宜居美丽肇庆。坚持环保准入“高门槛”，划定市域生态控制线，实施西江水质保护十五年规划，确保西江水质安全。坚持铁腕治理严要求，用三年左右的时间打赢大气综合治理攻坚战。坚持节能降耗低碳化，积极发展绿色经济，力争尽快达到国家生态文明建设示范市指标要求。

　　——补齐民生“短板”，让全市人民共享发展成果。持续加大民生投入，加快基本公共服务均等化，不断增进市民的获得感和幸福感。补齐公共产品服务供给不足的“短板”，构建现代国民教育体系和终身教育体系，推动居民健康水平与卫生服务指标达到省内中上水平，建成城市“十分钟文化圈”和农村“十里文化圈”。补齐城乡基本公共服务不均衡的“短板”，完善城乡一体化的社会保障体系，实现社会保障各项待遇水平与经济发展同步提升，实施农村基础设施改善工程，形成城乡一体的公共基础设施体系。补齐底线民生保障水平不高的“短板”，打好精准扶贫、精准脱贫三年攻坚战，确保到2018年全市4.3万贫困户、9.7万贫困人口全部脱贫。

　　本次大会审议通过《肇庆市国民经济和社会发展第十三个五年规划纲要》后，市政府将全力抓好“十三五”各项目标任务落实，确保全面小康蓝图如期变成现实，珠三角连接大西南枢纽门户城市地位不断巩固提升，开创肇庆更加幸福美好的明天！

扎实做好今年各项工作 努力实现“十三五”开门红

　　今年是全面建成小康社会决胜阶段的开局之年，经济社会发展主要预期目标是：地区生产总值增长8.5%左右，人均生产总值增长7.5%，一般公共预算收入增长5%，固定资产投资增长18%，规模以上工业增加值增长9.5%，社会消费品零售总额增长10.5%，实际吸收外资总额与上年持平，外贸出口总额增长2%，城乡居民收入增长与经济增长同步，居民消费价格指数涨幅控制在3%左右，城镇登记失业率控制在3%以内，人口出生率和自然增长率、节能减排和空气质量改善目标控制在省下达的计划以内。重点抓好以下十个方面工作：

　　一、全力以赴抓好稳增长

　　推进供给侧结构性改革攻坚。扎实推进“去产能、去库存、去杠杆、降成本、补短板”各项工作。实施传统支柱型产业转型升级技术路线和行动计划，改造提升新型建材、林产工业、金属加工业等传统优势产业，扶持一批五金企业向汽配生产转型。支持企业实施兼并重组盘活低效产能，促进现有产业和企业修复发展动力。加快城镇常住人口市民化，积极发展住房租赁市场，探索公租房货币化，推动保障房、安置房需求与商品房去库存之间有效衔接，加快房地产去库存，促进房地产业健康有序发展。强化金融风险排查防范，维护良好金融生态环境。打好降低企业制度性交易、人工、税费、社会保险、财务、物流等成本的“组合拳”，力争为企业减负46亿元。加快基础设施建设，增加公共产品和公共服务供给。

　　大力发展实体经济。抓好企业促建促产，支持企业扩张发展，力争新增规模以上工业企业50家、年主营业务收入超10亿元企业2家。推动“暖企行动”常态化，全面落实扶企惠企政策，实施企业稳岗补贴，清理规范涉企收费，严格规范中介服务收费。设立中小微企业应急转贷资金，力争企业贷款融资规模增长11%。

　　狠抓重点项目建设。实施三年滚动政府投资计划，今年安排计划投资467.2亿元的市重点项目100项（分解为180个子项目），抓紧启动计划投资169.2亿元的76个新开工项目，大力推进计划投资298亿元的104个续建项目。加大两级土地联合收储力度，力争全市新征收土地3万亩以上、盘活闲置用地3000亩。建立项目建设分类考核与财政扶持、用地安排挂钩激励办法。

　　挖掘出口和消费增长潜力。推进检验检疫通关一体化建设，支持企业拓展“一带一路”新兴出口市场，大力发展港口物流业和跨境电商，力促外贸结构优化、稳定增长。积极推进肇货全国行、网上行，继续抓好六大领域消费工程，拓展汽车、养老、家政、教育培训、文化体育等新兴消费，推动电子商务向农村和境外延伸，实施快递下乡工程，激活消费潜力，促进消费升级。

　　发挥金融支持实体经济重要作用。力争新增金融机构5家，银行业金融机构各项贷款增长20%以上。加强与政策性银行等金融机构合作，充分利用政府购买服务模式贷款业务和国家专项建设基金，加快推进棚户区改造、水利、城市地下综合管廊和体育场馆等项目建设。大力推广PPP模式，扩大引进社会资本。深化科技、产业、金融融合，开展“政银保”融资试点，加快发展普惠金融，发展股权投资和创业投资，新增8家上市企业，支持企业发行企业债、公司债、中期票据等直接融资。

　　二、打好市政交通建设大会战

　　打好市政建设大会战。启动中心城区市政交通重点项目21个，总投资约228亿元，其中端州城区实施10个项目，总投资72.3亿元；鼎湖城区实施4个项目，总投资11.1亿元；高要城区实施2个项目，总投资12.6亿元；肇庆新区实施5个项目，总投资132亿元。继续推进上年结转市政项目，确保完成阅江大桥主体工程，加快北岭路网升级改造、肇庆大桥扩建、国道321线城市化改造和鼎湖大道建设，启动江滨路升级改造工程。完善城区路网结构，缓解交通拥堵问题，五年投资58亿元，打通端州城区50条“瓶颈路”，其中今年投资25亿元启动建设19条，2017年和2018年分别启动建设18条、13条。

　　打好交通建设攻坚战。组织实施15项交通重点工程，计划投资123亿元。动工建设广佛肇高速广州石井至肇庆大旺段，加快广佛肇、汕昆、汕湛、怀阳高速肇庆段建设，确保广佛肇高速大旺至封开段年底前开通。加快实施肇佛交通对接的11项公路工程，启动高要回龙至金利快速路、大旺至三水北江大桥、国道321线民乐桥至三水交界段快速路和国道324线高要马安至高明一级公路改造建设。推进一批国省道扩建改造，谋划肇庆连接港珠澳大桥的高速公路。协调推进广茂铁路改造升级以及柳肇铁路、肇顺南城际轨道项目。高标准打造肇庆东站枢纽、市火车站综合枢纽，同步推进沿江沿路沿城轨综合开发。加快实施西江、北江航道肇庆段扩能升级工程，发展公水铁联运、江海联运。

　　三、全力大招商招大商

　　加快平台园区扩能增效。调整优化园区规划，制定实施支持通用厂房建设的优惠政策，高标准实施集中供电、供热、供冷、供气，积极盘活低效未利用的土地和厂房，力争全市产业园区新增工业用地不低于10平方公里，年内全市省级产业园区规模以上工业增加值实现277亿元。加快肇庆高新区开发建设，全年新开发土地面积2平方公里，积极推进31个在建工业项目和15个拟开工项目。创新粤桂合作特别试验区建设模式，抓紧推进拓展区开发建设。完成广佛肇经济合作区“一园一业、一主两辅”布局调整，新增产业项目投资16亿元。

　　提升招商选资质量水平。组建市招商委员会，强化落实招商层级责任和督查问责机制，实施产业专题招商、平台专业招商和小分队精准招商。建立全市统一的大招商路线图和招大商项目库，强化产业链招商和集群化招商，积极引进产业龙头项目和已孵化待转移的科技型项目，切实提高项目履约率、资金到位率和开工率，力争年内引进超20亿项目5个、超10亿项目10个。

　　四、加快现代产业集聚创新

　　以肇庆高新区为龙头引领全市科技协同创新。高标准编制实施高新区国家自主创新示范区三年创新创业计划和五年发展规划，大力吸引珠三角核心区高科技企业孵化成果落户转化，加快省微生物研究所工业技术转移中心等重点创新项目建设，力争实现国家级科技企业孵化器认定零的突破。发挥高新区引领带动作用，大力实施高新技术企业培育提升计划，重点扶持科技型中小微企业，力争全市新增高新技术企业24家、入库84家，高新技术产品产值占规上工业总产值比重达30.3%；实施产学研结合促进工程，扩大深化与高校和科研院所战略合作，引进发展一批新型研发机构，力争研发经费支出占GDP比重提高到1.3%，建成科技企业孵化器7家、在孵企业190家。

　　做大做强工业主导产业。抓好列入珠江西岸先进装备制造产业带规划的重点项目，大力引进汽车零部件、节能环保装备、智能制造装备等产业，推进肇庆高新区智能制造产业园、肇庆（高要）汽车零部件产业园等专业园区的建设招商，筹建智能制造公共服务平台，建设汽车零部件产业研究院，年内完成装备制造业投资212亿元。推动电子信息产业向高端环节延伸发展，培育发展新型电子材料、金属新材料、复合材料产业，积极发展工业生物、现代中药、动物疫苗等生物医药产业。推进中电四会、大唐高要热电冷联产项目，加快发展新能源产业。力争新一轮技术改造覆盖面扩大到30%，新增技改投资80亿元以上。

　　发展壮大现代服务业。大力发展生产性服务业，积极引进检验检测认证、科技投融资、节能环保等专业服务机构，培育发展电子商务、现代物流、大数据和服务外包产业。抓好肇庆电商谷等电商集聚园区建设，着力引进电商总部项目，发展玉器、五金、端砚、农副产品等特色电商平台。抓好端州睦岗片区现代流通综合改革，加快老城区存量工业用地向新产业载体转型，建设一批都市新业态产业集聚区。

　　积极发展旅游产业。编制实施星湖风景名胜区总体规划，建设鼎湖山新入口和七星岩北门游客服务中心，实施绿道升级行动计划，加快景区创5A步伐。加大旅游招商力度，启动端州区岩前休闲半岛旅游新业态项目建设，谋划推进府城复兴等旅游大项目。构筑全域旅游新格局，加强旅游标准化建设，拓展高铁经济带旅游合作，力争全市景区接待旅客人数突破3000万人次。

　　五、统筹协调推进城乡建设

　　全力推进肇庆新区建设发展。加快18平方公里起步区建设，抓好城市建设、棚户区改造、招商引资三大重点，扎实推进规划引领、政策运用、创新探索、投融资、土地征收、人力资源六大基础性保障工作。抓好总投资294亿元、年度投资75.2亿元的20项重点项目，未来三年完成市政设施和公共配套设施投资150亿元以上，建成鼎湖大道新区段及政文组团一期路网，实施城市地下综合管廊和同步建设工程以及砚阳调洪湖、长利涌、横槎涌水系综合整治工程，加快新区体育中心、中大附属肇庆医院、北大医疗康复医院、保利商务中心、肇庆东站站前商务区等项目建设。抓好投融资和土地征储，力争年内实现投融资237亿元，新增土地征收1.5万亩。以启迪环保科技城为龙头集聚发展节能环保产业，抓紧推进中国优质农产品交易中心等项目落地建设，加快打造环保科技、商贸会展、电子商务、健康医疗、高端电子“五个百亿产业组团”。

　　推动东南板块城市一体化建设。编制实施新一轮肇庆城市总体规划和一批专项规划，提升端州城区城市综合服务功能，以建设紫云新城、生态新城、江滨新城为重点拓展高要区城市发展空间，促进鼎湖—肇庆新区组团融合发展，推动肇庆高新区—大沙（四会）组团一体建设。强化跨江交通和东南板块内部的快速路建设，规划建设东南板块环城快速干线（端州睦岗至高要回龙、高要金利至四会大沙）及端州区与高新区、新区对接快线，谋划建设西江四桥（睦岗—大湾）。打造“一江两岸”景观带、大冲综合枢纽等一批城市主题地标，启动肇庆大桥以北滨江地区开发建设。优化布局端州区至肇庆新区、至高要区公交线路。

　　加快城镇化步伐。编制实施县域“三规合一”方案，提高村庄规划覆盖率。加快西江、绥江沿线城镇群扩容提质，推进山区县城新城区建设，抓好中心镇、重点镇和特色镇建设，把高要金利镇打造成为工业主导、产城互动的汽配五金特色专业镇。完善城镇基础设施和配套服务功能，提高综合承载能力，促进产城融合。深化户籍制度改革，加快实施居住证制度。

　　六、扎实做好“三农”工作

　　提升现代农业发展水平。认真落实今年中央1号文件精神。完成高标准基本农田和123宗水利设施项目建设任务，抓好24个国家级粮食创高产示范片建设，力争实现粮食“八连增”。全面推进农村土地承包经营权确权登记颁证工作，壮大新型农业经营主体，培育一批年销售收入超5亿元的大型骨干农业企业，打造一批农产品电商基地和省级休闲农业示范点。实施农业标准化生产，完善农产品质量安全可追溯体系和四级监管体系。扩大政策性农业保险覆盖面。稳步推进国有林场改革。

　　加快发展农村公共事业。抓好新一轮省级新农村连片示范工程和农村社区示范点建设，加快新一轮农村电网改造升级和县通镇公路升级改造，推进村村通自来水、通互联网工程。开展农村人居环境整治行动，实现农村生活垃圾集中收运处理体系常态化运营，再创一批省级卫生县城、卫生镇村、美丽乡村。关爱服务农村留守人员，推进农村社区法治建设，强化农村基层治理。大力帮扶老区开发建设。

　　集中力量打好脱贫攻坚战。加强精准扶贫识别工作，全面建档立卡，强化落实定点定单位对口帮扶责任，采取产业扶持、技能培训、转移就业、教育扶贫、保障兜底等方式，推进一户一策精准帮扶，形成全社会参与的扶贫大格局，确保脱贫攻坚不落下一个贫困地区、一个贫困群众，今年实现3.2万贫困人口脱贫，2017年和2018年分别实现4万、2.5万贫困人口脱贫。

　　七、打造宜居宜业宜游环境

　　构建绿色低碳生态环境。强化约束性指标管理，严格控制污染物排放总量，确保完成节能减排降碳年度任务。加强环境联合执法，坚决清理整顿环境违法违规项目，毫不松懈抓好大气污染重点领域整治，实行全市各地每月空气综合指标公布排名制度，确保全年空气质量优良天数达80%以上。启动羚山涌、石咀涌等城市黑臭水体整治，抓好端州城区雨污分流管网完善建设，推进西围涌、新窦涌等7条河涌综合整治，力争全市新建成污水处理厂12座，城镇污水处理率提高到87%。实施全民节能节水行动计划，积极推广新能源汽车。抓好四大林业重点生态工程，推动“森林进城、公园下乡”，建成羚羊峡古栈道森林公园、东调洪湖市民休闲公园，新增森林公园22个，确保创建成为国家森林城市。实行资源消耗和建设用地总量、强度“双控”，探索实施项目准入与投资强度、预期产出效益挂钩机制。推进矿产资源整合整顿。加强地质灾害防治，实施水土保持重点建设工程。

　　营造良好创业创新环境。深化行政审批制度改革，推进简政放权，进一步精简行政许可事项，全面推行投资准入负面清单、行政审批清单、政府监管清单“三单改革”，建立政府部门权责清单动态管理机制，切实做到“非禁即入”，消除阻碍创业创新壁垒。实施行政许可标准化，清理规范行政许可中介服务，提高行政许可效率。深化投融资体制改革，进一步放宽投资准入，激发投资创业热潮。加强创业创新公共服务，支持举办各类创业创新大赛，建设一批新兴产业“双创”示范基地，积极打造“肇梦空间—双创港”。

　　积极创建全国文明城市。深入开展主题文明创建活动，抓好“创文”八大专项整治，确保年度测评指标全部达标。进一步理顺市、区城市管理和综合执法关系，探索向社会购买服务的城市管理新模式，健全城市长效化、常态化、精细化管理机制。开展城市美化、绿化、亮化、数字化“四化”建设大行动，抓好城市市容环境卫生、户外广告和交通秩序整治。积极解决城区停车难和生活设施不配套等问题。

　　八、全面发展社会事业

　　创建广东省推进教育现代化先进市。两年内新建、扩建中小学校19所，今年新增学位6300个，完善异地务工人员随迁子女和引进人才子女入学机制，推动义务教育均衡优质标准化发展。支持肇庆学院加快高水平特色学科建设，推进肇庆医专和广东工商职业学院“升本”，积极争取国内外高等院校到肇办学，深化职业院校校企对接合作。继续实施第二期学前教育三年行动计划、特殊教育提升计划。促进老年教育事业发展。推动广宁县、封开县、德庆县创建成为广东省推进教育现代化先进县，确保我市创建成为广东省推进教育现代化先进市。

　　大力发展医疗卫生事业。完善城乡医疗卫生服务体系，深化公立医院综合改革，构建分级诊疗体系。加快市全科医生临床培养基地、市妇幼保健院科技楼等项目建设，加强县级医院关键医疗设备配备、专科特设岗位和住院医师规范化培训，推进乡镇卫生院业务用房改扩建和村卫生站“公建民营”规范化建设。提升重大疫情和疾病监测防控能力，促进基本公共卫生服务均等化。大力支持社会力量办医。擦亮肇庆中医药品牌。全面落实“二孩”政策，完成省下达的人口计划。

　　扎实推进文化建设。实施公共文化设施提升工程，加快市博物馆新馆、市文化发展中心和村（社区）综合文化服务中心建设，办好公益性群众文化活动。实施肇庆府学宫保护修缮工程和宋城保护与开发工程，推进古城墙保护和申报世界文化遗产工作，启动崇禧塔中西文化园景区扩建项目。扶持发展特色文化产业，大力引进文化产业项目。抓好第十五届省运会比赛场馆建设改造，办好第十届市运会，扩大公共体育场馆免费或低收费向社会开放，中心城区新建社区群众体育公园10个。

　　创新社会治理。深化村（居）务公开民主管理，抓好“五全”公共服务站建设。建成公共法律服务实体平台，加大信访、维稳和人民调解工作力度。完善“大视频”等社会治安防控体系，依法打击违法犯罪活动。加强食品药品安全和产品质量管理，严厉查处制售假冒伪劣商品违法行为。积极创建全国综合减灾示范社区和防震减灾示范市。强化落实安全生产“一岗双责”和企业主体责任，坚决防范和遏制安全事故发生。支持工青妇等群团组织参与社会治理，维护妇女儿童合法权益。加强国防动员和后备力量建设，积极创建全国双拥模范城。继续做好统计、民族、宗教、外事、侨务、港澳、对台、人防、地方志、档案、气象、水文、地震、老龄、残疾人、慈善等工作。

　　九、着力保障和改善民生

　　促进就业创业。办好公共就业服务平台，加强职业技能培训，统筹做好农村转移劳动力、高校毕业生和退役军人就业，重点帮扶城乡就业困难人员实现就业，实现新增城镇就业4万人、城镇失业人员再就业1.1万人。成立创业引导基金，实施大学生就业促进和创业引领计划，加大对妇女创业贷款贴息支持，力争新登记注册的初创企业户数、吸纳从业人员人数均增长10%以上。

　　提升社会保障水平。实施全民参保计划，加快社会保障城乡一体化。实施社保扩面提升工程，推进机关事业单位养老保险制度改革、企业全员足额参保、居民续保和应保尽保，继续提高退休人员基本养老金标准，确保各项社保待遇按时足额发放。完善基本医疗保险门诊统筹和异地就医结算制度，深化医保复合式付费方式改革，完善医疗救助“一站式”服务并与医保、大病保险有效衔接，实现大病保险全覆盖。城乡低保补助补差水平分别提高到418元和190元，继续提高孤儿、五保保障标准。加大棚户区改造和公共租赁住房供应力度，计划改造棚户区4790户、新增公共租赁住房712套（户），完成3311户农村危房改造任务。

　　继续办好惠民实事。市政府将在落实省十件民生实事有关任务的同时，集中力量办好今年十件惠民实事，提高底线民生保障水平，解决困难群体住房问题，改善农村生产生活条件，改善基层医疗卫生服务，促进教育资源公平均衡配置，促进创业带动就业，加强城乡生态宜居建设，加大环境污染整治力度，提升中心城区交通通行力，优化完善公共文体设施（具体内容见附件一）。

　　十、努力建设人民满意政府

　　强化法治政府建设。落实省创建珠三角法治政府示范区工作，严格在法律规定的范围内行使政府权力。自觉接受人大及其常委会法律监督和政协民主监督，定期向人大及其常委会报告工作和向政协通报情况，按时高质办好人大代表建议和政协提案。积极推进地方立法。完善行政机关重大决策合法性审查、重大行政决策听取意见机制以及听证、风险评估和实施后评估制度。强化行政执法监督，做好行政复议和行政应诉。启动“七五”普法。

　　强化责任政府建设。坚持法定职责必须为、法无授权不可为，对法定职责范围内的职能勇于负责，敢于担当，狠抓落实，切实提高政府部门执行力。建立政府部门行政问责、行政过错责任追究、责任倒查制度，强化对滥用权力、失职渎职的责任追究。加强行政绩效管理，突出整治懒政庸政怠政懈政等不作为、慢作为、乱作为现象。依法公开权力运行流程，主动接受群众监督。

　　强化效能政府建设。认真开展机关“严作风促实干年”活动，切实做到主动服务不推诿、协调服务不扯皮、高效服务不拖拉。优化部门职能设置，推动网上办事大厅向基层延伸服务，全面铺开基层公共服务综合平台建设，推广“一门式、一网式”服务管理，完善“一表制”和“三证合一”制度，力争让公众办事“零跑动”。创新事中事后监管方式，推行“双随机、一公开”监管。继续办好网络问政、《市民问政》，主动接受社会和舆论监督。

　　强化廉洁政府建设。认真贯彻《中国共产党廉洁自律准则》和《中国共产党纪律处分条例》，严格落实党风廉政建设主体责任，坚决从源头上防治腐败。深化政务公开，加强对政府性专项资金监管，落实政府投资重大项目廉政风险防控，推进审计全覆盖，实施党政主要领导干部和国有企业主要负责人任期经济责任审计，严格“三公”费用监管。坚持把纪律规矩挺在前面，强化行政监察，持之以恒纠正“四风”，坚决查处各类违法违纪案件。

　　各位代表！回望“十二五”，发展成绩令人振奋；展望“十三五”，新的征程任重道远。让我们紧密团结在以习近平同志为总书记的党中央周围，在省委、省政府和市委的正确领导下，深入实施“两区引领两化”战略，紧紧依靠全市人民，牢记使命，奋发有为，开拓进取，为率先全面建成小康社会、加快建设珠三角连接大西南枢纽门户城市而努力奋斗！
